# Supplementary material for: Ancestry and somatic profile indicate acral melanoma origin and prognosis
Source: Nature. 2026 Feb 18;651(8104):221–30. doi: 10.1038/s41586-025-09967-z (PMC12960246; doi:10.1038/s41586-025-09967-z)
Supplement: Supplementary file 1 — This file contains Supplementary Figs. 1–5, Tables 5, 6, 16, 22–27 and legends for Tables 1–4, 7–15, 17–21 and 28. [file 41586_2025_9967_MOESM1_ESM.pdf]

nature portfolio

https://doi.org/10.1038/s41586-025-09967-z

Supplementary information

Ancestry and somatic profile indicate acral melanoma origin and prognosis

In the format provided by the authors and unedited

Supplementary Information

Ancestry and somatic profile indicate acral melanoma origin and prognosis

Patricia Basurto-Lozada[1,2], Martha Estefania Vázquez-Cruz[1], Christian Molina-Aguilar[1], Amanda Jiang[3-5], Dekker C. Deacon[3,4], Dennis Cerrato-Izaguirre[6], Irving Simonin-Wilmer[1], Fernanda G. Arriaga-González[1,7], Kenya L. Contreras-Ramírez[1], Emiliano Ferro-Rodríguez[1], Jamie Billington[7], Eric T. Dawson[8], J. Rene C. Wong-Ramirez[1,9], Johana Itzel Ramos-Galguera[1], Alethia Álvarez-Cano[10], Dorian Y. García-Ortega[11], O. Isaac García-Salinas[1,7], Alfredo Hidalgo-Miranda[12], Mireya Cisneros-Villanueva[12], Peter A. Johansson[13,14], Héctor Martínez-Said[11], Pilar Gallego-García[15], Mark J. Arends[16], Ingrid Ferreira[7], Mark Tullett[17], Rebeca Olvera-León[1,7], Louise van der Weyden[7], Martín del Castillo Velasco-Herrera[7], Rodrigo Roldán-Marín[18], Helena Vidaurri de la Cruz[19], Luis Alberto Tavares-de-la-Paz[20], Diego Hinojosa-Ugarte[21], Rachel L. Belote[22], D. Timothy Bishop[23], Marcos Díaz-Gay[15,24-26], Ludmil B. Alexandrov[24-26], Yesennia Sánchez-Pérez[6], Gino K. In[27], Richard M. White[28], Patrícia A. Possik[7,29], Robert L. Judson-Torres[3-5], David J. Adams[7], Carla Daniela Robles-Espinoza[1,7,*]

1. Laboratorio Internacional de Investigación sobre el Genoma Humano, Universidad Nacional Autónoma de México, Santiago de Querétaro, Mexico, 76230
2. Posgrado en Ciencias Biológicas, Universidad Nacional Autónoma de México, Unidad de Posgrado, Edificio D, 1° Piso, Circuito de Posgrados, Ciudad Universitaria, Coyoacán, C.P. 04510, CDMX, México
3. Huntsman Cancer Institute, University of Utah Health Sciences Center, Salt Lake City, Utah, USA
4. Department of Dermatology, University of Utah, Salt Lake City, UT, USA
5. Department of Oncological Sciences, University of Utah, Salt Lake City, UT, USA
6. Subdirección de Investigación Básica, Instituto Nacional de Cancerología (INCan), San Fernando No. 22, Tlalpan, Ciudad de México CP. 14080, Mexico.
7. Wellcome Sanger Institute, Hinxton, Cambridgeshire, CB10 1SA, UK.
8. Nvidia Corporation, Santa Clara, CA, USA.
9. Research Program in Systems Oncology, Research Programs Unit, Faculty of Medicine, University of Helsinki, Helsinki, Finland.
10. Surgical Oncology, Christus Muguerza Alta Especialidad, Monterrey, Nuevo Leon, Mexico.
11. Surgical Oncology, Skin, Soft Tissue & Bone Tumors Department, National Cancer Institute, Mexico City, Mexico.
12. Laboratorio de Genómica del Cáncer, Instituto Nacional de Medicina Genómica (INMEGEN), Mexico City, Mexico.
13. QIMR Berghofer, Brisbane, QLD, Australia
14. University of Queensland, Brisbane, QLD, Australia
15. Digital Genomics Group, Cancer Genomics Program, Spanish National Cancer Research Center (CNIO), Madrid, Spain
16. Edinburgh Pathology, Cancer Research UK Scotland Centre, Institute of Genetics and Cancer, University of Edinburgh, Edinburgh EH4 2XU, UK
17. Department of histopathology, University Hospitals Sussex, St Richard hospital, Spitalfield lane, Chichester
18. Dermato-Oncology Clinic, Research Division, Faculty of Medicine, Universidad Nacional Autónoma de México, Mexico City, Mexico.
19. Pediatric Dermatology Service, Hospital General de México Dr. Eduardo Liceaga, Ministry of Health. Mexico City, Mexico.
20. Surgical Oncology, Bajio Regional High Specialty Hospital, Leon, Mexico.
21. Division of Surgery, Instituto Nacional de Ciencias Médicas y Nutrición Salvador Zubirán. México City, México.
22. The Ohio State University, Department of Molecular Genetics, Columbus, Ohio, United States
23. Leeds Institute of Medical Research, University of Leeds, Leeds, UK.
24. Department of Cellular and Molecular Medicine, University of California San Diego, La Jolla, CA, USA.
25. Department of Bioengineering, University of California San Diego, La Jolla, CA, USA.
26. Moores Cancer Center, University of California San Diego, La Jolla, CA, USA.
27. University of Southern California, Keck School of Medicine, Norris Comprehensive Cancer Center, Division of Oncology, Los Angeles, CA, USA.
28. Nuffield Department of Medicine, Ludwig Institute for Cancer Research, University of Oxford, Oxford, UK
29. Division of Basic and Experimental Research, Brazilian National Cancer Institute, Rua Andre Cavalcanti 37, Rio de Janeiro, RJ, 20231-050, Brazil.

***Correspondence to:** Carla Daniela Robles-Espinoza, drobles@liigh.unam.mx

Supplementary Information Guide

Supplementary Figure 1 ………………………………………… Page 3

Supplementary Figure 2 ………………………………………… Page 4

Supplementary Figure 3 ………………………………………… Page 5

Supplementary Figure 4 ………………………………………… Page 6

Supplementary Figure 5 ………………………………………… Page 7

Supplementary Table 1 (legend only, supplemented as separate file) ………… Page 8

Supplementary Table 2 (legend only, supplemented as separate file) ………… Page 8

Supplementary Table 3 (legend only, supplemented as separate file) ………… Page 8

Supplementary Table 4 (legend only, supplemented as separate file) ………… Page 8

Supplementary Table 5 ………………………………………… Page 9

Supplementary Table 6 ………………………………………… Page 10

Supplementary Table 7 (legend only, supplemented as separate file) ………… Page 11

Supplementary Table 8 (legend only, supplemented as separate file) ………… Page 11

Supplementary Table 9 (legend only, supplemented as separate file) ………… Page 11

Supplementary Table 10 (legend only, supplemented as separate file) ………… Page 11

Supplementary Table 11 (legend only, supplemented as separate file) ………… Page 11

Supplementary Table 12 (legend only, supplemented as separate file) ………… Page 11

Supplementary Table 13 (legend only, supplemented as separate file) ………… Page 11

Supplementary Table 14 (legend only, supplemented as separate file) ………… Page 11

Supplementary Table 15 (legend only, supplemented as separate file) ………… Page 11

Supplementary Table 16 ………………………………………… Page 12

Supplementary Table 17 (legend only, supplemented as separate file) ………… Page 13

Supplementary Table 18 (legend only, supplemented as separate file) ………… Page 13

Supplementary Table 19 (legend only, supplemented as separate file) ………… Page 13

Supplementary Table 20 (legend only, supplemented as separate file) ………… Page 13

Supplementary Table 21 (legend only, supplemented as separate file) ………… Page 13

Supplementary Table 22 ………………………………………… Page 14

Supplementary Table 23 ………………………………………… Page 15

Supplementary Table 24 ………………………………………… Page 16

Supplementary Table 25 ………………………………………… Page 17

Supplementary Table 26 ………………………………………… Page 18

Supplementary Table 27 ………………………………………… Page 19

Supplementary Table 28 (legend only, supplemented as separate file) ………… Page 20

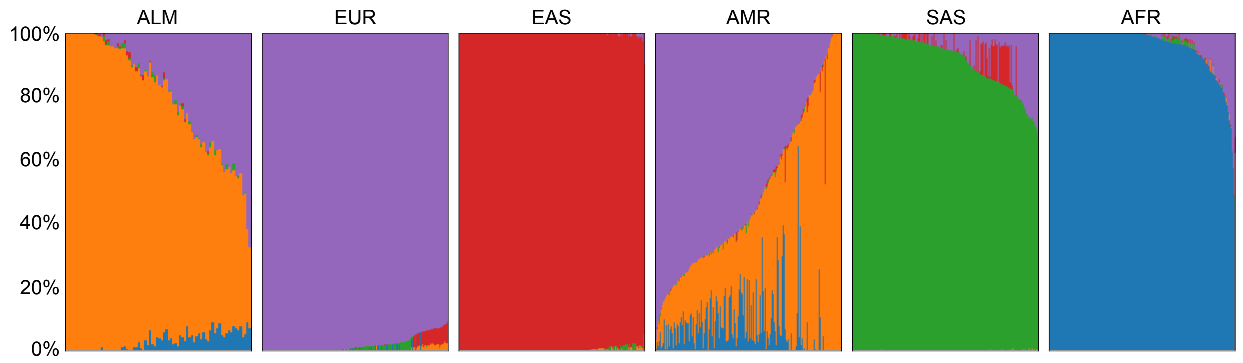

Supplementary Figure 1. Estimation of ancestry proportions per sample together with the superpopulations of the 1000 Genomes dataset. The leftmost panel corresponds to the samples genotyped in this study (n=80). The following panels correspond to the superpopulations in the 1000 Genomes Project. Five superpopulations are plotted, corresponding to African (AFR, blue), American (AMR, orange), South Asian (SAS, green), East Asian (EAS, red), and European (EUR, purple).

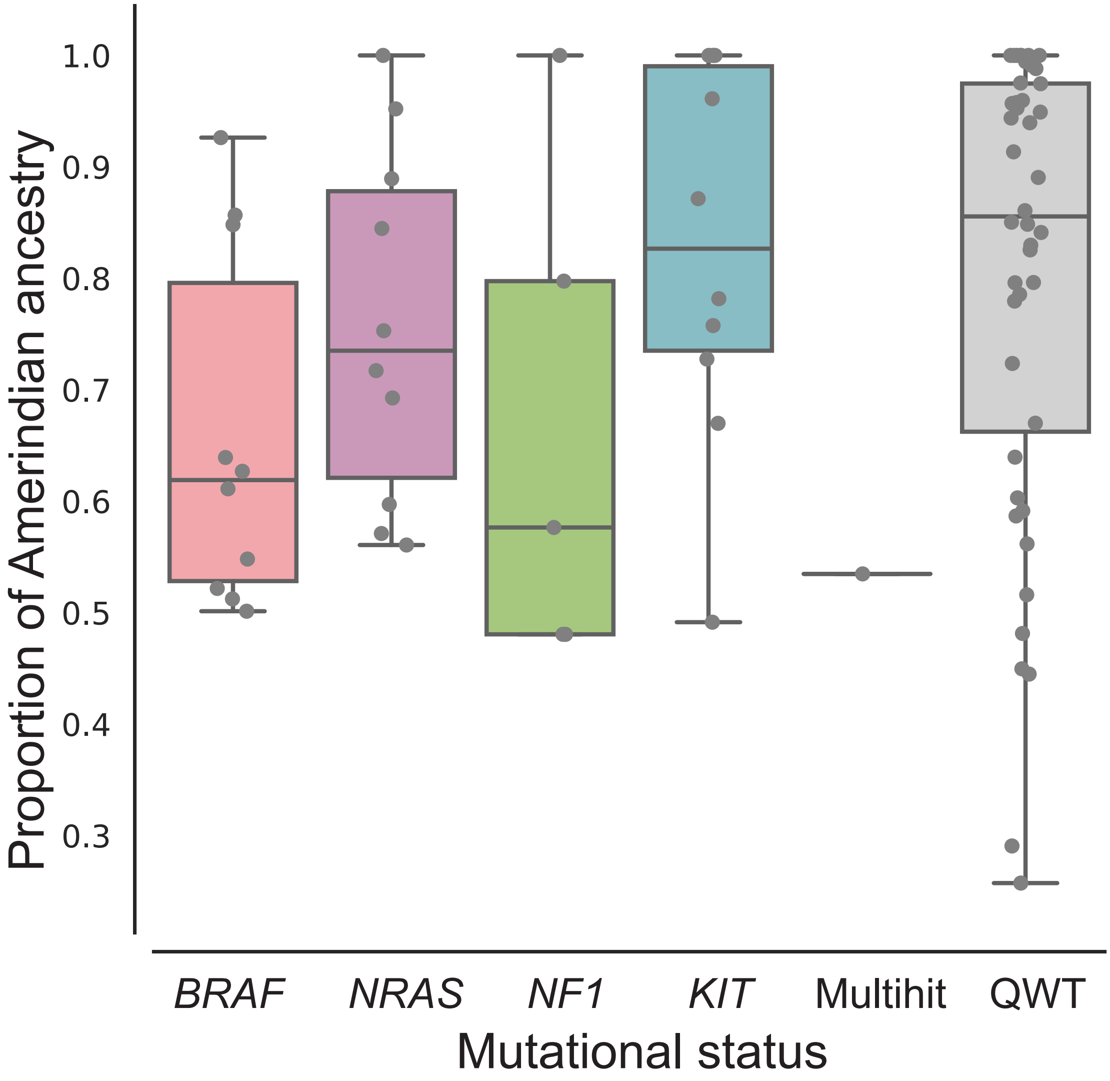

Supplementary Figure 2. Boxplot of the proportion of Amerindian ancestry among patients classified by genomic subtype. Each dot corresponds to a sample. The central line within each box represents the median value, the box boundaries represent the interquartile range (IQR), and the whiskers extend to the lowest or highest data point still within 1.5xIQR.

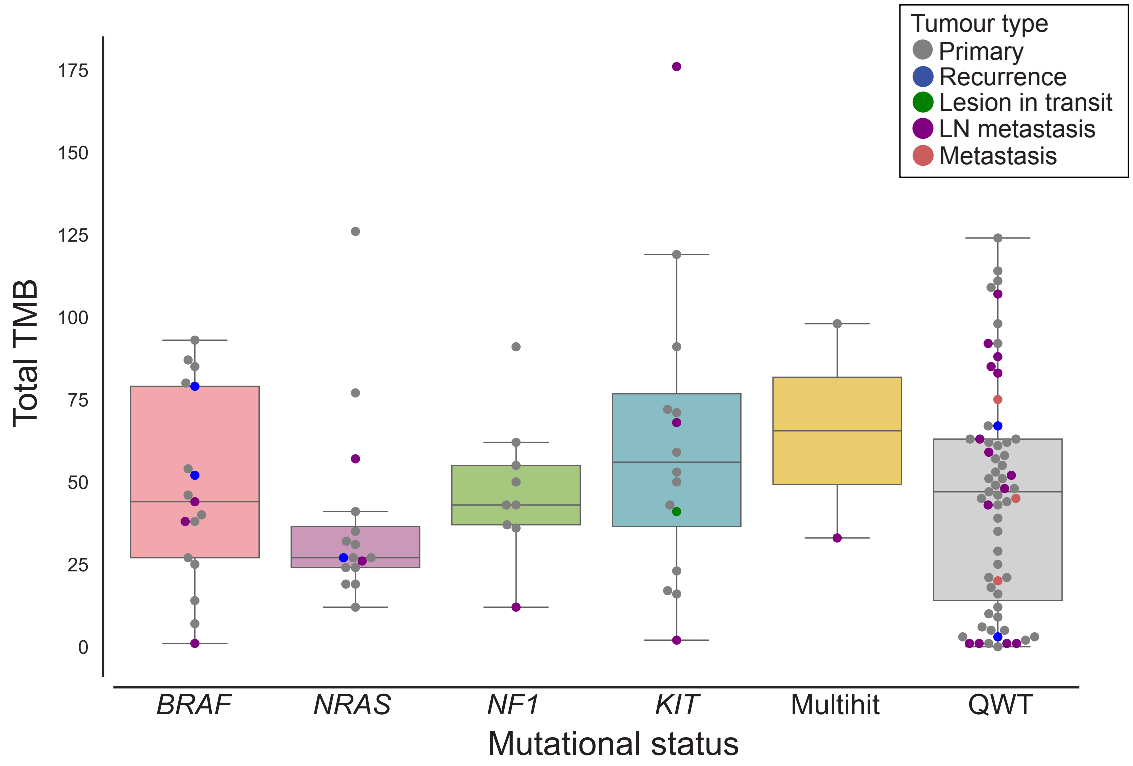

Supplementary Figure 3. Boxplot of total TMB for all samples classified by genomic subtype. Each dot corresponds to a sample, and colours represent tumour type. The central line within each box represents the median value, the box boundaries represent the interquartile range (IQR), and the whiskers extend to the lowest or highest data point still within 1.5xIQR.

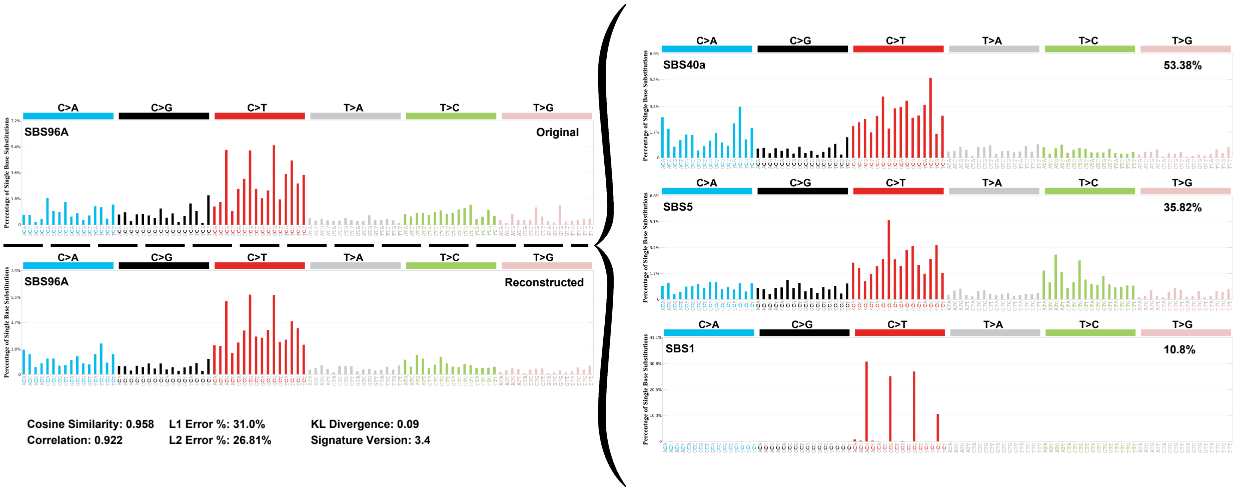

Supplementary Figure 4. Decomposition plot of single-base substitution mutational signatures for all acral melanoma samples. Samples that had mutation data (116) were included in this analysis.

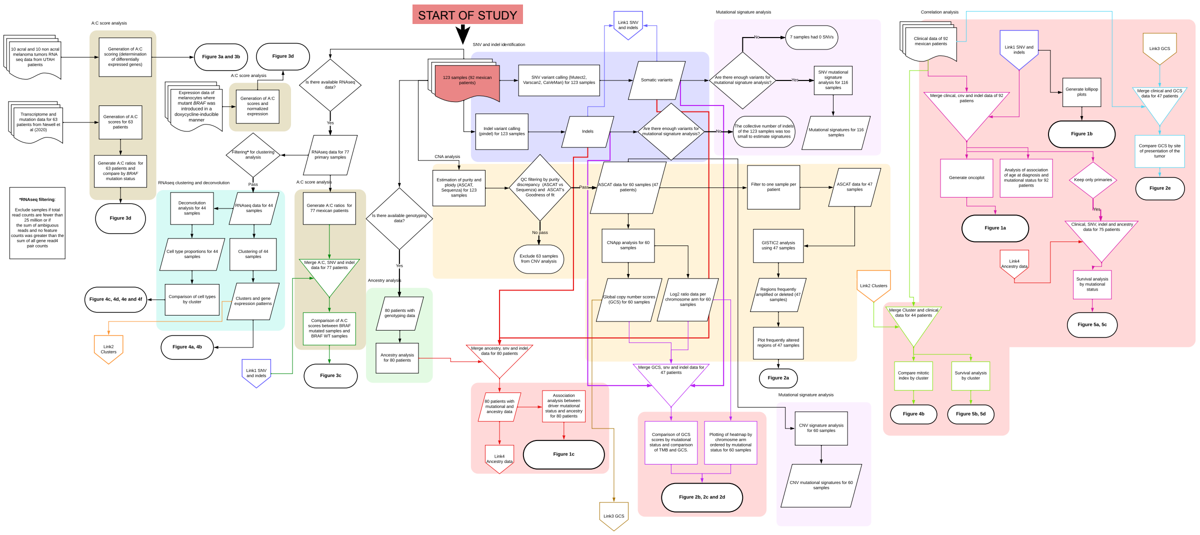

Supplementary Figure 5. Flowchart describing all analyses and steps in this work. Different colours represent different sections of the study, the resulting main figure from each analysis is also indicated in the text.

Supplementary Tables

Supplementary Table 1. Clinical and molecular information for patients and samples analysed in this study. Clinical information, along with genomic subtype and classification, total TMB, copy number alteration counts, transcriptomic cluster and socioeconomic status are included. For self-reported socioeconomic status, the range is 1-7, with 1 being the lowest and 7 being the highest. The value of Breslow thickness and ulceration for metastatic samples is derived from its corresponding primary.

To facilitate reading, this table is provided as a separate Excel file. Patient ages have been rounded down to the nearest 5-year interval, and all dates are reported as month/year to preserve anonymity. Access to the complete dataset is available upon request and subject to Data Access Committee (DAC) approval.

Supplementary Table 2. Ancestry proportions for five superpopulations for samples in this study and 1000 Genomes Project samples. The header is labelled with the inferred population from comparison with the 1000 Genomes projects.

To facilitate reading, this table is provided as a separate Excel file.

Supplementary Table 3. dNdScv results of neutrality tests at the gene level. This table includes information on the number of substitutions of each class observed in each gene, maximum-likelihood estimates of dN/dS ratios for each gene, *P*-values obtained by Likelihood-Ratio Tests as described in (Martincorena et al, 2017)[64] and q-values obtained by Benjamini-Hodgberg's multiple testing correction.

To facilitate reading, this table is provided as a separate Excel file.

Supplementary Table 4. *TERT* promoter sequencing results. In the "Amplification column", 0 = not amplified, 1 = low amplification, 2 = high amplification, LQ = low quality. TERT status classification: 1=mutated, 0=wild type.

To facilitate reading, this table is provided as a separate Excel file.

Supplementary Table 5. Analysis of clinical, molecular and transcriptional characteristics by sex. Two percentages are shown in each cell, the first one is by column and second one by row.

Characteristic		Female (n=54) (100%) (58%)	Male (n=38) (100%) (42%)	Total (100%)
Age	Mean	60.79	61.82	61.21
	Range	32-98	43-85	32-98
Breslow depth	Mean	5.98	7.48	6.61
	Range	0.45-50 (n=52)	0.6-38 (n=37)	0.45-50
TMB	Mean	50.57	45.05	48.29
	Range	0-176	1-124	0-176
Stage	0	1 (1.85%) (100%)	0 (0%) (0%)	1 (100%)
	I	8 (14.81%) (80%)	2 (5.26%) (20%)	10 (100%)
	II	14 (25.93%) (56%)	11 (28.95%) (44%)	25 (100%)
	III	26 (48.15%) (55%)	21 (55.26%) (46%)	47 (100%)
	IV	5 (9.26%) (55%)	4 (10.53%) (45%)	9 (100%)
Primary location	Foot	42 (77.78%) (55%)	34 (89.47%) (45%)	76 (100%)
	Hand	4 (7.4%) (100%)	0 (0%) (0%)	4 (100%)
	Subungual	8 (14.81%) (66%)	4 (10.53%) (34%)	12 (100%)
Classification	*BRAF*	10 (18.52%) (83%)	2 (5.26%) (17%)	12 (100%)
	NRAS	10 (18.52%) (83%)	2 (5.26%) (17%)	12 (100%)
	NF1	3 (5.56%) (43%)	4 (10.53%) (57%)	7 (100%)
	KIT	9 (16.67%) (75%)	3 (7.89%) (25%)	12 (100%)
	Multihit	1 (1.85%) (100%)	0 (0%) (0%)	1 (100%)
	Driver mutated	33 (61%) (75%)	11 (29%) (25%)	44 (100%)
	QWT	21 (38.89%) (44%)	27 (71.05%) (56%)	48 (100%)
		**Female (n= 25) (100%) (56%)**	**Male (n = 19) (100%) (44%)**	**44 (100%)**
RNAseq Clusters (n=44)	Cluster1	8 (32%) (57%)	6 (31.57%) (43%)	14 (100%)
	Cluster2	9 (36%) (56%)	7 (36.84%) (56%)	16 (100%)
	Cluster3	8 (32%) (57%)	6 (31.57%) (43%)	14 (100%)
Sample mean coverage		45.05	44.56	44.86

Supplementary Table 6. Patients with multiple samples and their driver mutational status. For the analysis mentioned in the text, patients with two primaries sequenced were not taken into account, as these are often two fragments of the same primary.

Patient	Classification	Primary	Primary 2	LN metastasis	LN metastasis 2	Recurrence	Lesion in transit	Other
PD40961	*BRAF*	V600E	V600E			V600E		
PD40965	*KIT*	K642E				No driver		K642E
PD40966	*KIT*	K642E		K642E	No driver			
PD40967	QWT	No driver		No driver				
PD40969	QWT	No driver		No driver				
PD40971	QWT	No driver		No driver				
PD40978	QWT	No driver		No driver				
PD40980	QWT	No driver				No driver		
PD40983	*KIT*	K642E		No driver			K642E	
PD40986	QWT	No driver		*NF1* focal deletion				
PD40987	*BRAF*	V600E		V600E				
PD41002	*NRAS*	Q61R		Q61R		Q61R		
PD41020	*NRAS*	Q61L		Q61L/*NF1* focal deletion				
PD41025	*NRAS*	Q61R		No driver				
PD41035	QWT	No driver	No driver					
PD41039	*KIT*	K642E		K642E				
PD41043	*BRAF*	V600E		V600E				
PD41046	QWT	No driver		No driver	No driver			
PD41910	*KIT*	R634Q		No driver				
PD41913	*NRAS*	Q61R	Q61R					
PD41915	*BRAF*	V600E		V600E				
PD41920	QWT	No driver		No driver				
PD41923	QWT	No driver		No driver				
PD51928	*NRAS*	G12R		G12R				
PD51969	*NF1*	Y1292*	Y1292*					

Supplementary Table 7. Amplification and deletion peaks found in acral melanoma samples.

To facilitate reading, this table is provided as a separate Excel file.

Supplementary Table 8. Cytobands, q values, location and genes contained within amplification peaks.

To facilitate reading, this table is provided as a separate Excel file.

Supplementary Table 9. Cytobands, q values, location and genes contained within deletion peaks.

To facilitate reading, this table is provided as a separate Excel file.

Supplementary Table 10. Amplification and deletion peaks found in QWT acral melanoma samples.

To facilitate reading, this table is provided as a separate Excel file.

Supplementary Table 11. Cytobands, q values, location and genes contained within amplification peaks in QWT samples.

To facilitate reading, this table is provided as a separate Excel file.

Supplementary Table 12. Cytobands, q values, location and genes contained within deletion peaks in QWT samples.

To facilitate reading, this table is provided as a separate Excel file.

Supplementary Table 13. Amplification and deletion peaks found in driver-mutated acral melanoma samples.

To facilitate reading, this table is provided as a separate Excel file.

Supplementary Table 14. Cytobands, q values, location and genes contained within amplification peaks in driver-mutated samples.

To facilitate reading, this table is provided as a separate Excel file.

Supplementary Table 15. Cytobands, q values, location and genes contained within deletion peaks in driver-mutated samples.

To facilitate reading, this table is provided as a separate Excel file.

Supplementary Table 16. List of candidate genes from acral and cutaneous melanoma datasets.

Housekeeping Genes	Acral Melanocyte Enriched Genes	Cutaneous Melanocyte Enriched Genes
RPL19	*SPINT2*	*RPL15*
RPLP0	*IFFO2*	*SNRPD2*
TUBB	*IRX3*	*NENF*
POLR2A	*ID1*	*CRYL1*
POLR1B	*PDLIM7*	*COX6C*
TBP	*REC8*	*GNG2*
	WNT4	*PSMB4*
	IRX5	*COX4I1*
	CCND2	*DAD1*
	ZSWIM4	*TMEM147*
	SERTAD1	*TMBIM4*
	MEG3	*RPS27L*
	NTRK2 pan	*COX8A*
	ID2	*UQCR11*
	ID3	*RAC1*
	RAB3B	*OST4*
	NAV2	*SRP14*
	MEG3	*TIMP1*
	IGDCC4	*PLP1*
	PDLIM3	*HPGD*
	FRZB	*AKAP12*
	PHACTR3	*MCOLN3*
	CDR1	*ASAH1*
	HOXA13	*IFITM3*
	HOXB13	*APOE*
	HOXC13	*SLITRK2*
	HOXD13	*APCDD1*
	TBX4	*CST3*
	DLX4	*SEMA5A*
	HAND2	*LGI3*
	IGF2	*CALM1*
	ANK1	*HOXB5*
	SLIT1 and SLIT3	*HOXB6*
	AATK	*HOXB7*
	PTK2	*HOXB8*
	WWP2	*HOXB9*
	EGFL7	*KRT15*
	ACADVL	
	MEST	

Supplementary Table 17. Gene module identification and mean gene expression value per gene per RNA cluster.

To facilitate reading, this table is provided as a separate Excel file.

Supplementary Table 18. Gene enrichment analysis for genes in Gene Module 2 (M2), which has the highest expression in Cluster 1.

To facilitate reading, this table is provided as a separate Excel file.

Supplementary Table 19. Gene enrichment analysis for genes in Gene Module 1 (M1), which has the highest expression in Cluster 2.

To facilitate reading, this table is provided as a separate Excel file.

Supplementary Table 20. Gene enrichment analysis for genes in Gene Module 1 (M3), which has the highest expression in Cluster 3.

To facilitate reading, this table is provided as a separate Excel file.

Supplementary Table 21. RNA deconvolution results per sample.

To facilitate reading, this table is provided as a separate Excel file.

Supplementary Table 22. Multivariate logistic regression to test the association of any driver mutation on recurrence-free survival.

Num. of observations		73				
LR chi2(6)		18.11				
Prob > chi2		0.006				
Pseudo R2		0.1791				
Log likelihood		-41.484649				
**Recurrence**	**Odds ratio**	**Std. Err**	**z**	**P>|z|**	**[95% Conf. Interval]**	
Any mutation	5.310556	3.325634	2.67	0.008	1.556293	18.12127
Sex	0.9844121	0.5578017	-0.03	0.978	0.3242328	2.988801
Age at diagnosis	1.016565	0.0244662	0.68	0.495	0.9697256	1.065667
Tumour stage	3.545433	1.973259	2.27	0.023	1.191027	10.554
Date of diagnosis	1.000076	0.000339	0.23	0.822	0.9994121	1.000741
Ancestry (AMR)	21.06773	33.44283	1.92	0.055	0.9384777	472.9461
Intercept	0.0018219	0.0129485	-0.89	0.375	1.63E-09	2042.56

Supplementary Table 23. Multivariate logistic regression to test the association of cluster assignment on recurrence-free survival.

Num. of observations		44				
LR chi2(6)		9.3				
Prob > chi2		0.1573				
Pseudo R2		0.1562				
Log likelihood		-25.116653				
**Recurrence**	**Odds ratio**	**Std. Err**	**z**	**P>|z|**	**[95% Conf. Interval]**	
Cluster 2 (Cluster 1 reference)	6.683601	6.598269	1.92	0.054	0.9653317	46.2748
Cluster 3 (Cluster 1 reference)	2.373717	2.045131	1	0.316	0.4385876	12.84699
Sex	1.24812	0.9355723	0.3	0.767	0.2872155	5.42381
Age at diagnosis	0.9963314	0.0305672	-0.12	0.905	0.9381865	1.05808
Tumour stage	2.591876	1.867251	1.32	0.186	0.6315211	10.63752
Date of diagnosis	1.000321	0.0006131	0.52	0.6	0.9991203	1.001524
Intercept	0.0000775	0.000994	-0.74	0.461	9.22E-16	6502608

Supplementary Table 24. Log-rank test for homogeneity, any mutation on overall survival.

Any mutation	Events observed	Events expected
no	4	7.43
yes	11	7.57
Total	15	15
chi2(1)	3.13	
Pr>chi2	0.0766	

Supplementary Table 25. Log rank test of homogeneity, mutation classification on overall survival.

Mutation	Events observed	Events expected
QWT	4	7.43
BRAF	4	2.02
KIT	1	1.85
Multihit	1	0.05
NF1	2	1.17
NRAS	3	2.48
Total	15	15
chi2(5)	21.88	
Pr>chi2	0.0006	

Supplementary Table 26. Cox proportional hazards analysis testing the association of any mutation on overall survival.

Num. of subjects	73		Num. of observations		73	
Num. of failures	13		LR chi2(6)		11.16	
Time at risk	83920		Prob > chi2		0.0483	
Log likelihood	-38.181793					
**Variable**	**Hazard ratio**	**Std. Err**	**z**	**P>|z|**	**[95% Conf. Interval]**	
Any mutation	3.185216	2.253241	1.64	0.101	0.796148	12.74336
Sex	1.573245	0.9712176	0.73	0.463	0.4691595	5.275602
Age at diagnosis	1.027321	0.032233	0.86	0.39	0.9660492	1.09248
Tumour stage	4.051354	2.897533	1.96	0.05	0.9972859	16.45813
Ancestry (AMR)	0.0445928	0.0713461	-1.94	0.052	0.0019382	1.025987

Supplementary Table 27. Chi squared test of independence, cluster assignment and survival,

	Death		Total
Cluster	no	yes	
1	14	0	14
	100%	0%	100%
2	9	7	16
	56.25%	43.75%	100%
3	11	3	14
	78.57%	21.43%	100%
Pearson chi2(2)	8.1576	Pr	0.017
likelihood-ratio chi2(2)	10.6862	Pr	0.005
Cramer's V	0.4306		
Gamma	-0.4344	ASE	0.29
Kendall's tau-b	-0.2265	ASE	0.158

Supplementary Table 28. Comparison between ASCAT and Sequenza purity and ploidy estimates. Samples in black font passed all QC.

To facilitate reading, this table is provided as a separate Excel file.
