# Supplementary material for: Ancestry and somatic profile indicate acral melanoma origin and prognosis
Source: Nature. 2026 Feb 18;651(8104):221–30. doi: 10.1038/s41586-025-09967-z (PMC12960246; doi:10.1038/s41586-025-09967-z)
Supplement: Supplementary file 2 — Reporting Summary [file 41586_2025_9967_MOESM2_ESM.pdf]

nature portfolio

Corresponding author(s): Robles-Espinoza, C.D.

Last updated by author(s): Nov 7, 2025

Reporting Summary

Nature Portfolio wishes to improve the reproducibility of the work that we publish. This form provides structure for consistency and transparency in reporting. For further information on Nature Portfolio policies, see our Editorial Policies and the Editorial Policy Checklist.

Statistics

For all statistical analyses, confirm that the following items are present in the figure legend, table legend, main text, or Methods section.

n/a	Confirmed	
☐	☒	The exact sample size (*n*) for each experimental group/condition, given as a discrete number and unit of measurement
☐	☒	A statement on whether measurements were taken from distinct samples or whether the same sample was measured repeatedly
☐	☒	The statistical test(s) used AND whether they are one- or two-sided<br>*Only common tests should be described solely by name; describe more complex techniques in the Methods section.*
☐	☒	A description of all covariates tested
☐	☒	A description of any assumptions or corrections, such as tests of normality and adjustment for multiple comparisons
☐	☒	A full description of the statistical parameters including central tendency (e.g. means) or other basic estimates (e.g. regression coefficient) AND variation (e.g. standard deviation) or associated estimates of uncertainty (e.g. confidence intervals)
☐	☒	For null hypothesis testing, the test statistic (e.g. F, t, r) with confidence intervals, effect sizes, degrees of freedom and P value noted<br>*Give P values as exact values whenever suitable.*
☒	☐	For Bayesian analysis, information on the choice of priors and Markov chain Monte Carlo settings
☒	☐	For hierarchical and complex designs, identification of the appropriate level for tests and full reporting of outcomes
☐	☒	Estimates of effect sizes (e.g. Cohen's *d*, Pearson's *r*), indicating how they were calculated

Our web collection on statistics for biologists contains articles on many of the points above.

Software and code

Policy information about availability of computer code

Data collection	Clinical data was collected using Microsoft Excel v16.
Data analysis	ADMIXTURE v1.3.0, ancestry estimation<br>PLINK v1.9, quality control and merging acral dataset with 1000 Genome Project for ancestry estimation<br>BWA-mem v0.7.17-r1188, sequencing read alignment<br>samtools v1.9, sequencing data analysis<br>bcftools v1.9, vcf file manipulations<br>fastqc v0.11.3, sequencing data quality control<br>GATK v4.2.3.0, genome analysis toolkit including Mutect2 v4.1.0.0, tools for quality, variant calling and data filtering<br>maftools v2.2.10, visualisation of somatic mutation data<br>Conpair v0.2, sample concordance estimation<br>Varscan2 v2.3.9, somatic variant calling<br>dNdScv v0.0.1.0, driver gene identification<br>Sequenza v3.0.0, somatic copy number estimation<br>GISTIC2 v2.0.23, genomic identification of significantly altered regions<br>CNApp , analysis and comparison of copy number alterations<br>SigProfiler v1.1.24, mutational signature identification and analysis<br>STAR 2.5.0c, transcriptome sequencing read alignment<br>DESeq2 v1.44.0, differential gene expression<br>limma v3.64.1, batch effect correction<br>nSolver Analysis Software, normalisation of NanoString data

Prism v10.2.1, plotting software
cola R 2.10.0, consensus clustering of transcriptome data
EPIC v1.1.7, transcriptome data deconvolution
cgpCaVEMan (v1.15.2), for mutation calling
SmartPhase (v1.2.1), for mutation calling together with cgpCaVEMan
cgpPindel (v.3.10.0), for indel calling
ASCAT (v3.1.2), for copy number calling
cnvkit 0.9.10, for copy number estimation
STATA 19.5, for survival analyses
What Is My Melanocytic Signature (WIMMS), https://wimms.tanlab.org
clusterProfiler (v4.12.6), for functional enrichment analysis
org.Hs.eg.db (3.19.1), for gene annotation

Python and R were used to perform the different bioinformatic analyses. Scripts used for analysis are at https://github.com/CGBio-Lab/Mex-acral-exomes-transcriptomes

For manuscripts utilizing custom algorithms or software that are central to the research but not yet described in published literature, software must be made available to editors and reviewers. We strongly encourage code deposition in a community repository (e.g. GitHub). See the Nature Portfolio guidelines for submitting code & software for further information.

Data

Policy information about availability of data

All manuscripts must include a data availability statement. This statement should provide the following information, where applicable:

- Accession codes, unique identifiers, or web links for publicly available datasets
- A description of any restrictions on data availability
- For clinical datasets or third party data, please ensure that the statement adheres to our policy

Sequencing data are available at the European Genome-Phenome Archive (EGA). DNA sequencing data are available under ENA accession number EGAD00001015755 and RNA sequencing data under ENA accession number EGAD00001015756. The 1000 Genomes Project datasets can be dowloaded from https://www.internationalgenome.org/data. The GRCh38 reference genome can be downloaded from https://www.ncbi.nlm.nih.gov/datasets/genome/GCF_000001405.40/. Sequencing data for the Newell. et al study is available from the European Genome-Phenome Archive (EGA) under study accession EGAS00001001552 and dataset accession EGAD00001005500. Access to the data can be gained through application to the Data Access Committee for the dataset. Information on how to apply for access is available at the EGA dataset link: https://ega-archive.org/datasets/EGAD00001005500. The Cancer Genome Atlas (TCGA) Skin Cutaneous Melanoma Firehose Legacy cohort data can be downloaded from cBioPortal (https://www.cbioportal.org/). RNA sequencing data from NcNeil et al is available from GEO under accession number GSE150849.

Research involving human participants, their data, or biological material

Policy information about studies with human participants or human data. See also policy information about sex, gender (identity/presentation), and sexual orientation and race, ethnicity and racism.

Reporting on sex and gender	Samples for both sexes were included in this study. No gender (identity/presentation) or sexual orientation information is presented.
Reporting on race, ethnicity, or other socially relevant groupings	Throughout this manuscript, we use the term ''ancestry' to refer to genetically inferred ancestry (through whole-genome genotyping and mapping onto the 1000 Genomes superpopulations). The socioeconomic status data is self-reported, as is indicated in the manuscript. No mentions to race or ethnicity, or other social groupings of the participants, is made.
Population characteristics	All this information is provided in the manuscript, briefly, the median age of diagnosis was 60 years of age, 58.7% of participants were female, with the majority of tumours diagnosed Stage III (51%), ulcerated (68%), and in the feet (82.6%). Most patients (90%) had predominantly Amerindian ancestry (median 81%).
Recruitment	Consenting and recruitment of patients started in 2017 and ended in 2019. Because of the challenges of recruiting significant numbers of participants with AM, patients diagnosed in earlier years who were still attending follow-up clinics were recruited. This fact is taken into account in the survival analyses.
Ethics oversight	The protocol for sample collection was approved by the Mexican National Cancer Institute's (Instituto Nacional de Cancerología, INCan, México) Ethics and Research committees (017/041/PBI;CEI/1209/17) and the United Kingdom's National Health Services (NHS, UK) (18/EE/00076).

Note that full information on the approval of the study protocol must also be provided in the manuscript.

Field-specific reporting

Please select the one below that is the best fit for your research. If you are not sure, read the appropriate sections before making your selection.

☒ Life sciences ☐ Behavioural & social sciences ☐ Ecological, evolutionary & environmental sciences

For a reference copy of the document with all sections, see nature.com/documents/nr-reporting-summary-flat.pdf

Life sciences study design

All studies must disclose on these points even when the disclosure is negative.

Sample size	Sample size was determined by all patients that fulfilled the inclusion criteria and signed an informed consent form between 2017 and 2019. Inclusion criteria were:<br>1. Being treated at the National Cancer Institute of Mexico with a histological diagnosis of acral melanoma,<br>2. Patients which have samples available for analysis: Slides or paraffin blocks with enough material for diagnosis and sequencing, and/or the presence of primary or metastatic tumour which is accessible for sampling,<br>3. Patients that have signed the informed consent form for diagnosis, treatment and follow-up, and<br>4. Patients whose data had sufficient quality for follow-up analyses.
Data exclusions	Samples were excluded when not enough nucleic acids where available after extraction for DNA or RNA sequencing. Samples were also excluded if paired samples failed concordance or there was presence of contamination as stated in the text. The rationale behind this exclusion was to just include samples were somatic variant calling and RNAseq analysis could be performed. For copy number analysis, samples with discrepant results between ASCAT and Sequenza tools, or whose goodness of fit was lower than 95 were excluded to keep results only from samples with high confidence copy number estimates.
Replication	The analyses belonging to the transcriptional score calulcations were replicated once in an independent dataset (Newell et al, 2020). For the cell culture experiments for assessing gene expression, three biological replicates were conducted for each condition. All other analyses replication was not performed as this is a descriptive, observational study.
Randomization	No randomisation was done as this is an observational study. This study had mainly a descriptive approach (observational study), without any interventions. As the cancer type limited the samples available, all samples that met criteria were included.
Blinding	Blinding was not applicable as this study was observational with a descriptive approach.

Reporting for specific materials, systems and methods

We require information from authors about some types of materials, experimental systems and methods used in many studies. Here, indicate whether each material, system or method listed is relevant to your study. If you are not sure if a list item applies to your research, read the appropriate section before selecting a response.

Materials & experimental systems

n/a	Involved in the study	
☒	☐	Antibodies
☒	☐	Eukaryotic cell lines
☒	☐	Palaeontology and archaeology
☒	☐	Animals and other organisms
☒	☐	Clinical data
☒	☐	Dual use research of concern
☒	☐	Plants

Methods

n/a	Involved in the study	
☒	☐	ChIP-seq
☒	☐	Flow cytometry
☒	☐	MRI-based neuroimaging

Plants

Seed stocks	*Report on the source of all seed stocks or other plant material used. If applicable, state the seed stock centre and catalogue number. If plant specimens were collected from the field, describe the collection location, date and sampling procedures.*
Novel plant genotypes	*Describe the methods by which all novel plant genotypes were produced. This includes those generated by transgenic approaches, gene editing, chemical/radiation-based mutagenesis and hybridization. For transgenic lines, describe the transformation method, the number of independent lines analyzed and the generation upon which experiments were performed. For gene-edited lines, describe the editor used, the endogenous sequence targeted for editing, the targeting guide RNA sequence (if applicable) and how the editor was applied.*
Authentication	*Describe any authentication procedures for each seed stock used or novel genotype generated. Describe any experiments used to assess the effect of a mutation and, where applicable, how potential secondary effects (e.g. second site T-DNA insertions, mosiacism, off-target gene editing) were examined.*
